# Effect of food intake on 92 biomarkers for cardiovascular disease

**DOI:** 10.1371/journal.pone.0178656

**Published:** 2017-06-06

**Authors:** Magnus Dencker, Ylva Gårdinger, Ola Björgell, Joanna Hlebowicz

**Affiliations:** 1 Department of Medical Imaging and Physiology, Skåne University Hospital, Lund University, Malmö, Sweden; 2 Department of Cardiology, Skåne University Hospital, Lund University, Lund, Sweden; Maastricht University, NETHERLANDS

## Abstract

**Objective:**

The present study evaluates the effect of food intake on 92 biomarkers for cardiovascular disease (CVD).

**Methods:**

Twenty two healthy subjects (11 male and 11 female aged 25.9±4.2 years) were investigated. A total of 92 biomarkers were measured before a standardized meal as well as 30 and 120 minutes afterwards with the Proseek Multiplex CVD III kit.

**Results:**

The levels for eight biomarkers decreased significantly (P<0.05) 30 minutes after food intake. The levels for seven biomarkers remained significantly decreased 120 minutes after food intake. Nine biomarker decreased significantly at 120 minutes after food intake. The changes were between 4–30%, most commonly around 5%. Only six biomarkers showed a difference of 10% or more due to food intake. The biggest differences were observed for Insulin-like growth factor-binding protein 1 (30%); Azurocidin, Cystatin-B, and Myeloperoxidase (13%); Monocyte chemotactic protein 1 (11%); and Myeloblastin (10%), all 120 minutes after food intake.

**Conclusions:**

This study shows that food intake affects several different CVD biomarkers, but the effect is predominantly modest. Timing of blood sampling in relation to food intake, therefore, appears not to be a major concern. Further studies are warranted in older healthy subjects and in patients with various cardiac diseases to determine whether the findings are reproducible.

## Introduction

Cardiovascular disease (CVD) is the leading cause of death in the world. Current risk assessment systems are far from perfect [1,2]. There is a continuous search for novel biomarkers that could improve the risk assessment in CVD [3–9]. Digestion of food is known to have significant hemodynamic and metabolic effects [10–15], and may, therefore, affect different biomarkers. It is relevant from a practical point of view to investigate whether these biomarkers are affected by food intake, as the timing of blood sampling would then become relevant. Moreover, it could be of physiological interest to investigate biological responses to food intake. The present study evaluates the effect of food intake in healthy volunteers on 92 different emerging biomarkers for CVD. To the best our knowledge, this has not been done before.

## Materials and methods

### Study population

The study investigated 22 healthy Caucasians (11 male and 11 female, aged 25.9±4.2 years). None of the subjects had a prior history or showed any symptoms of cardiovascular disease or any other chronic diseases. None of the subjects were taking any cardiovascular medication. Height and weight were measured and BMI and body surface area [16] were calculated. The subjects ingested a standardized meal consisting of 300 g rice pudding (AXA Goda Gröten Risgrynsgröt; Lantmännen AXA, Järna, Sweden). The total caloric value of the meal was 330 kcal: 10% from protein (9 g), 58% from carbohydrates (48 g), and 32% from fat (12 g). Our ambition with the meal was to mimic a breakfast. Written informed consent was obtained from all participants. The trial is registered in the US National Library of Medicine with the trial registration number NCT01027507. The study was approved by the regional ethical review board in Lund, Sweden.

### Blood samples

Blood samples were collected before the meal as well as 30 and 120 minutes afterwards and then frozen. No beverages were consumed during the experiment. One of the blood samples collected 30 minutes after the meal was defective and excluded from the analysis. The 91 biomarkers were analyzed by the Proximity Extension Assay technique using the Proseek Multiplex CVD III 96x96 reagents kit (Olink Bioscience, Uppsala, Sweden) as previously described [17,18]. Data are presented as arbitrary units (AU). Values can be transformed to actual concentrations using transformation algorithms on the Olink Bioscience website (www.olink.com). The conversion, however, is not exact. The vast majority of measurements for N-terminal prohormone brain natriuretic peptide failed to reach the detection level. The Proseek Multiplex CVD III kit has primarily been designed for heart failure with elevated N-terminal prohormone brain natriuretic peptide levels. The samples are therefore diluted 1:100, and this could explain why the vast majority of measurements for N-terminal prohormone brain natriuretic peptide failed to reach the detection level. The Proseek Multiplex Metabolism kit, where the samples are only diluted 1:10, were therefore used to detect the levels of N-terminal prohormone brain natriuretic peptide (Olink Bioscience, Uppsala, Sweden). One sample for Matrix extracellular phosphoglycoprotein, three samples for Spondin-1, and four samples for Chitotriosidase-1 failed to reach detection levels. In these cases the values were set at the detection levels. The 92 biomarkers that were analyzed are as follows (intra- and inter-assay variation): Tumor necrosis factor receptor superfamily member 14 (8%,10%), Low-density lipoprotein receptor (8%,9%), Integrin beta-2 (8%,11%), Interle ukin-17 receptor A (8%,10%), Tumor necrosis factor receptor 2 (8%,10%), Matrix metalloproteinase-9 (8%,12%), Ephrin type-B receptor 4 (8%,9%), Interleukin-2 receptor subunit alpha (8%,8%), Osteoprotegerin (8%,11%), CD166 antigen (7%,8%), Trefoil factor 3 (8%,9%), P-selectin (8%,10%), Cystatin-B (8%,9%), Monocyte chemotactic protein 1 (8%,12%), Scavenger receptor cysteine-rich type 1 protein M130 (7%,9%), Galectin-3 (8%,9%), Granulins (7%,11%), Matrix extracellular phosphoglycoprotein (9%,12%), Bleomycin hydrolase (8%,11%), Perlecan (7%,9%), Lymphotoxin-beta receptor (8%,10%), Neurogenic locus notch homolog protein 3 (9%,10%), Metalloproteinase inhibitor 4 (9%,12%), Contactin-1 (7%,9%), Cadherin-5 (11%,12%), Trem-like transcript 2 protein (8%,12%), Fatty acid-binding protein, adipocyte (8%,9%), Tissue factor pathway inhibitor (9%,12%), Plasminogen activator inhibitor 1 (8%,10%), C-C motif chemokine 24 (9%,13%), Transferrin receptor protein 1 (6%,9%), Tumor necrosis factor receptor superfamily member 10C (7%,10%), Growth/differentiation factor 15 (9%,11%), E-selectin (7%,10%), Azurocidin (7%,8%), Protein delta homolog 1 (8%,11%), Spondin-1 (8%,12%), Myeloperoxidase (7%,8%), C-X-C motif chemokine 16 (9%,12%), Interleukin-6 receptor subunit alpha (8%,9%), Resistin (7%,13%), Insulin-like growth factor-binding protein 1 (8%,10%), Chitotriosidase-1 (8%,11%), Tartrate-resistant acid phosphatase type 5 (7%,10%), C-C motif chemikine 22 (8%,15%), Pulmonary surfactant-associated protein D (9%,9%), Elafin (8%,13%), Epithelial cell adhesion molecule (8%,11%), Aminopeptidase N (7%,8%), Tyrosine-protein kinase receptor UFO (8%,11%), Interleukin-1 receptor type 1 (8%,10%), Matrix metalloproteinase-2 (9%,13%), Tumor necrosis factor receptor superfamily member 6 (8%,12%), Myoglobin (8%,15%), Tumor necrosis factor ligand superfamily member 13B (8%,12%), Myeloblastin (8%,14%), Proprotein convertase subtilisin/kexin type 9 (10%,25%), Urokinase plasminogen activator surface receptor (8%,10%), Osteopontin (8%,11%), Cathepsin D (7%,10%), Peptidoglycan recognition protein 1 (8%,12%), Carboxypeptidase A1 (7%,10%), Junctional adhesion molecule A (8%,11%), Galectin-4 (8%,10%), Interleukin-1 receptor type 2 (8%,10%), Tyrosine-protein phosphatase non-receptor type substrate 1 (8%,9%), C-C motif chemokine 15 (9%,15%), Caspase-3 (9%,16%), Urokinase-type plasminogen activator (8%,14%), Carboxypeptidase B (7%,12%), Chitinase-3-like protein 1 (8%,10%), ST2 protein (8%,10%), Tissue-type plasminogen activator (9%,16%), Secretoglobin family 3A member 2 (10%,22%), Epidermal growth factor receptor (7%,10%), Insulin-like growth factor-binding protein 7 (9%,13%), Complement component C1q receptor (8%,11%), Interleukin-18-binding protein (8%,10%), Collagen alpha-1(I) chain (6%,10%), Paraoxonase (9%,13%), Cathepsin Z (7%,9%), Matrix metalloproteinase-3 (9%,14%), Retinoic acid receptor responder protein 2 (9%,11%), Intercellular adhesion molecule 2 (8%,11%), Kallikrein-6 (8%,11%), Platelet-derived growth factor subunit A (9%,15%), Tumor necrosis factor receptor 1 (8%,12%), Insulin-like Growth Factor-Binding Protein 2 (9%,15%), von Willebrand factor (8%,12%), Platelet endothelial cell adhesion molecule (7%,10%), C-C motif chemokine 16 (10%,18%), and N-terminal prohormone of brain natriuretic peptide (4%,6%).

### Statistical analysis

Data are presented as mean ± standard deviation (SD), and the difference ± SD (for the difference) at 30 and 120 minutes after food intake. Statistical analyses were carried out using Statistica 12 (StatSoft Inc, Tulsa, OK, USA). Comparison between fasting values versus 30 and 120 minutes after food intake for any given biomarker was analyzed for significance with repeated measures ANOVA with Post hoc analysis by Tukey's test. Statistical significance was set at P<0.05.

## Results and discussion

Table 1 displays descriptive statistics of the study population. The levels for eight biomarkers decreased significantly 30 minutes after food intake. The levels for seven biomarkers remained significantly decreased 120 minutes after food intake. Nine biomarker decreased significantly at 120 minutes after food intake. The changes were between 4–30%, most commonly around 5%. Only six biomarkers showed a difference of 10% or more due to food intake. The biggest differences were observed for Insulin-like growth factor-binding protein 1 (30%); Azurocidin, Cystatin-B, and Myeloperoxidase (13%); Monocyte chemotactic protein 1 (11%); and Myeloblastin (10%), all 120 minutes after food intake. A summary of the findings is in Table 2. Statistical significance is reported in three different levels; *(P<0.05), **(P<0.01), and ***(P<0.001). The values between P<0.05 and P<0.01 should be treated with caution because of the possibility of mass significance.

**Table 1:** Subjects’ anthropometric characteristics. Values are mean ± SD.

Variable	
Sex (male/female)	11/11
Body mass (kg)	69±10
Height (cm)	177±8
BMI (kg/m^2^)	21.8±2.2
BSA (m^2^)	1.8±0.2

**Abbreviations**: Body mass index (BMI). Body surface area (BSA).

**Table 2:** Summary of findings for 92 biomarkers before, and the differences 30 and 120 minutes after a standardized meal. All values are in arbitrary units (Mean±SD). *Indicates significant difference (P<0.05), ** (P<0.01), and ***(P<0.001), compared to fasting values.

Variable	Fasting (n = 22)	Difference 30 minutes after food intake (n = 21)	Difference 120 minutes after food intake (n = 22)
Tumor necrosis factor receptor superfamily member 14	4.61±0.36	-0.31±0.19*	-0.36±0.61**
Low-density lipoprotein receptor	2.60±0.49	-0.05±0.16	0.03±0.52
Integrin beta-2	5.03±0.42	-0.15±0.22	-0.16±0.58
Interleukin-17 receptor A	2.67±0.49	-0.14±0.15	-0.10±0.55
Tumor necrosis factor receptor 2	4.12±0.43	-0.16±0.17	-0.11±0.61
Matrix metalloproteinase-9	5.55±0.52	-0.22±0.32	-0.34±0.59*
Ephrin type-B receptor 4	1.05±0.31	-0.01±0.13	-0.02±0.43
Interleukin-2 receptor subunit alpha	3.28±0.42	-0.11±0.16	-0.04±0.56
Osteoprotegerin	2.22±0.30	-0.05±0.20	0.01±0.53
CD166 antigen	4.48±0.35	-0.11±0.18	-0.02±0.63
Trefoil factor 3	5.45±1.38	-0.13±0.23	-0.08±0.60
P-selectin	9.30±0.61	-0.34±0.30*	-0.48±0.69**
Cystatin-B	5.11±1.06	-0.29±1.15	-0.65±1.29*
Monocyte chemotactic protein 1	3.44±0.41	-0.30±0.28*	-0.33±0.68**
Scavenger receptor cysteine-rich type 1 protein M130	5.87±0.63	-0.13±0.15	-0.04±0.58
Galectin-3	4.95±0.43	-0.09±0.23	-0.13±0.65
Granulins	5.82±0.31	-0.10±0.16	-0.04±0.54
Matrix extracellular phosphoglycoprotein	1.70±0.69	-0.13±0.21	-0.13±0.56
Bleomycin hydrolase	5.95±0.61	-0.01±0.33	-0.13±0.72
Perlecan	5.66±0.38	-0.00±0.36	-0.07±0.62
Lymphotoxin-beta receptor	2.65±0.36	-0.14±0.19	-0.06±0.59
Neurogenic locus notch homolog protein 3	2.32±0.59	-0.15±0.18	-0.06±0.60
Metalloproteinase inhibitor 4	2.60±0.49	-0.16±0.20	0.04±0.64
Contactin-1	2.04±0.37	-0.10±0.17	-0.02±0.57
Cadherin-5	2.44±0.42	-0.12±0.19	-0.03±0.62
Trem-like transcript 2 protein	4.87±0.44	-0.26±0.26	-0.29±0.73
Fatty acid-binding protein, adipocyte	4.05±0.62	-0.05±0.18	-0.04±0.65
Tissue factor pathway inhibitor	7.63±0.51	-0.06±0.22	0.12±0.61
Plasminogen activator inhibitor 1	7.65±0.47	-0.13±0.17	-0.03±0.51
C-C motif chemokine 24	5.21±0.87	-0.09±0.19	-0.02±0.63
Transferrin receptor protein 1	3.68±0.70	-0.10±0.11	-0.07±0.33
Tumor necrosis factor receptor superfamily member 10C	4.90±0.63	-0.10±0.15	-0.06±0.53
Growth/differentiation factor 15	3.35±0.46	-0.14±0.20	-0.07±0.62
E-selectin	2.62±0.55	-0.09±0.14	-0.04±0.52
Azurocidin	6.46±1.01	-0.48±0.50*	-0.86±0.83***
Protein delta homolog 1	4.78±0.78	-0.15±0.17	-0.07±0.61
Spondin-1	0.46±0.24	-0.08±0.16	-0.03±0.38
Myeloperoxidase	4.99±0.65	-0.37±0.38*	-0.67±0.57***
C-X-C motif chemokine 16	4.66±0.34	-0.13±0.20	-0.06±0.60
Interleukin-6 receptor subunit alpha	10.84±0.39	-0.10±0.17	-0.02±0.56
Resistin	6.34±0.63	-0.26±0.22	-0.40±0.66**
Insulin-like growth factor-binding protein 1	4.40±1.05	-0.15±0.47	-1.32±0.77***
Chitotriosidase-1	4.46±1.40	-0.21±0.18	-0.25±0.62
Tartrate-resistant acid phosphatase type 5	2.87±0.57	-0.20±0.25	-0.09±0.65
C-C motif chemokine 22	3.31±1.28	-0.12±0.17	-0.04±0.57
Pulmonary surfactant-associated protein D	1.69±0.70	-0.12±0.22	-0.01±0.49
Elafin	4.46±0.57	-0.11±0.21	-0.12±0.58
Epithelial cell adhesion molecule	5.10±1.02	-0.10±0.20	-0.15±0.61
Aminopeptidase N	4.44±0.40	-0.09±0.14	-0.03±0.54
Tyrosine-protein kinase receptor UFO	6.92±0.49	-0.10±0.16	-0.02±0.61
Interleukin-1 receptor type 1	5.68±0.39	-0.13±0.18	-0.03±0.61
Matrix metalloproteinase-2	3.53±0.48	-0.16±0.18	-0.08±0.65
Tumor necrosis factor receptor superfamily member 6	4.21±0.43	-0.12±0.15	-0.06±0.60
Myoglobin	5.93±0.80	-0.05±0.21	-0.08±0.67
Tumor necrosis factor ligand superfamily member 13B	5.62±0.39	-0.11±0.15	-0.01±0.59
Myeloblastin	7.02±0.82	-0.42±0.39*	-0.74±0.83***
Proprotein convertase subtilisin/kexin type 9	0.82±0.30	-0.10±0.19	-0.04±0.45
Urokinase plasminogen activator surface receptor	4.49±0.46	-0.16±0.24	-0.28±0.61*
Osteopontin	3.48±0.55	-0.02±0.17	0.14±0.64
Cathepsin D	4.55±0.34	-0.17±0.21	-0.06±0.59
Peptidoglycan recognition protein 1	7.58±0.61	-0.26±0.25	-0.42±0.66**
Carboxypeptidase A1	4.55±0.52	-0.05±0.22	0.14±0.62
Junctional adhesion molecule A	4.56±0.32	-0.11±0.93	-0.41±0.65*
Galectin-4	2.14±0.46	-0.18±0.21	-0.24±0.58
Interleukin-1 receptor type 2	4.55±0.48	-0.12±0.16	-0.03±0.57
Tyrosine-protein phosphatase non-receptor type substrate 1	2.80±0.34	-0.09±0.14	-0.02±0.57
C-C motif chemokine 15	6.03±0.49	-0.16±0.18	-0.11±0.62
Caspase-3	6.66±0.69	0.02±1.00	-0.60±0.86*
Urokinase-type plasminogen activator	4.35±0.48	-0.22±0.18*	-0.19±0.56
Carboxypeptidase B	3.38±0.55	-0.04±0.23	0.19±0.64
Chitinase-3-like protein 1	6.47±0.44	-0.21±0.20	-0.31±0.60*
ST2 protein	2.98±0.80	-0.05±0.15	0.10±0.58
Tissue-type plasminogen activator	4.57±0.76	-0.16±0.17	-0.15±0.66
Secretoglobin family 3A member 2	3.10±0.82	-0.18±0.16	-0.15±0.54
Epidermal growth factor receptor	2.67±0.35	-0.10±0.15	-0.03±0.54
Insulin-like growth factor-binding protein 7	3.39±0.57	-0.20±0.22	-0.08±0.76
Complement component C1q receptor	8.76±0.37	-0.11±0.16	-0.04±0.50
Interleukin-18-binding protein	4.66±0.43	-0.12±0.18	-0.03±0.60
Collagen alpha-1(I) chain	3.71±0.56	-0.11±0.18	0.00±0.45
Paraoxonase (PON 3)	6.30±0.82	-0.10±0.19	-0.00±0.63
Cathepsin Z	4.57±0.37	-0.13±0.19	0.00±0.59
Matrix metalloproteinase-3	5.98±0.88	-0.33±0.21*	-0.45±0.63***
Retinoic acid receptor responder protein 2	10.64±0.26	-0.12±0.14	-0.05±0.45
Intercellular adhesion molecule 2	4.26±0.40	-0.14±0.17	-0.04±0.62
Kallikrein-6	5.80±0.34	-0.02±0.20	-0.05±0.59
Platelet-derived growth factor subunit A	5.62±0.71	-0.20±0.21	-0.05±0.66
Tumor necrosis factor receptor 1	5.79±0.39	-0.15±0.21	-0.14±0.56
Insulin-like Growth Factor-Binding Protein 2	6.81±0.89	-0.09±0.16	0.11±0.57
von Willebrand factor	6.56±0.46	-0.17±0.30	-0.25±0.74
Platelet endothelial cell adhesion molecule	4.43±0.39	-0.08±0.69	-0.23±0.60
C-C motif chemokine 16	6.02±0.46	-0.13±0.20	-0.04±0.65
N-terminal prohormone of brain natriuretic peptide	0.69±0.61	-0.03±0.22	0.11±0.47

To our knowledge, this is the first study to evaluate the effect of food intake on plasma proteins measured by the Proseek Multiplex CVD III kit. This investigation showed that 17 of the 92 investigated biomarkers were affected by food intake. The changes were, however, modest. Only six biomarkers showed a difference of 10% or more due to food intake. The changes were, for the most part, around 5%. The observed effects of food intake are approximately half of the intra- and interassay variation which are about 10% for most of the biomarkers. This suggests that the need to standardize food intake is not generally necessary when using this kit (Proseek Multiplex CVD III). There were, however, some exceptions. The biggest differences were observed for Insulin-like growth factor-binding protein 1 (30%); Azurocidin, Cystatin-B, and Myeloperoxidase (13%); Monocyte chemotactic protein 1 (11%); and Myeloblastin (10%), all 120 minutes after food intake. Insulin-like growth factor-binding protein 1 has been shown to predict cardiovascular mortality and morbidity in patients with acute myocardial infarction [19,20]. The level of insulin-like growth factor-binding protein has been shown to regulate insulin-like growth factor-I bioactivity, glucose homeostasis, and tissue regeneration, and increases during inflammation [21]. It is known that insulin suppresses the insulin-like growth factor-binding protein 1 expression [22]. This may explain why we observed a significantly lower level of insulin-like growth factor-binding protein 1 in the postprandial period in our study. It could, however, be difficult in the acute setting to standardize food intake, but our findings clearly suggest that this should be done when investigating Insulin-like growth factor-binding protein 1. Inflammation has been suggested to be the underlying pathogenesis of a range of the most common cardiovascular diseases [23,24]. Myeloblastin is a protease that degrades proteins such as elastin, fibronectin, laminin and collagen and has mainly been investigated in vasculitis [25]. Azurocidin, also known as cationic antimicrobial protein CAP37 or heparin-binding protein is an important multifunctional inflammatory mediator [26]. Myeloperoxidase has also been shown to be an inflammatory marker in coronary heart disease [24]. A postprandial increase in white blood cell and platelet counts and a decrease in the release of myeloperoxidase from polymorphonuclear leukocyte has previously been observed [27]. In our study, we also observed a decreased Myeloperoxidase level in the postprandial period. Both Cystatin-B, and Monocyte chemotactic protein 1 have both recently been investigated in large population studies and have been associated with an increased incidence of CVD [5,9]. The difference of 10–13% for Myeloblastin, Cystatin-B, Monocyte chemotactic protein 1, Azurocidin and Myeloperoxidase suggests that not standardizing food intake prior to sampling may introduce additional error.

Recent technological advances makes it possible to measure multiple plasma proteins simultaneously. In the current study the Proseek Multiplex CVD III kit was used to simultaneously measure 92 plasma proteins. The proteins have been selected because of their importance in cardiovascular disease. According to the manufacturer, 28 of the biomarkers are inflammatory/immune system, 25 exploratory, 21 CVD, 11 metabolic and 6 CVD/Inflammation. This technology has recently been used in a multitude of studies [3–9], and our investigation adds methodological information in this developing field.

We are aware of only one study that has evaluated the impact of food intake on the biomarkers in the present study. Jahn and co-workers evaluated the effect of food intake on 26 of the 91 biomarkers in the present study [28]. This study evaluated the biomarker levels before and 240 minutes after a high-fat breakfast in 16 healthy and 18 metabolic syndrome subjects. Their findings were partly at odds with the present investigation. The findings in the study by Jahn et al. and in our study were concordant for 11 biomarkers (Growth differentiation factor-15, TNF receptor 1, Interleukin-6 receptor subunit alpha, TNF-receptor 2, FAS receptor, ST2 protein, Cathepsin D, Integrin beta binding protein 2, Spondin-1, Epidermal growth factor recptor, N-terminal prohormone-BNP), whereas it was not for 15 biomarkers. It is interesting to observe that Jahn et al. found no change in 7 biomarkers where we did (Cystatin B, Monocyte chemotactic protein 1, TNF-receptor superfamily member 14, Myloperoxidase, Resistin, Chitinase-3-like protein 1, Urokinase-type plasminogen activator receptor), and the opposite was also found for 8 biomarkers (Matrix metalloproteinase-3, Galectin-3, E-selectin, Osteoprotegerin, Kallikrein-6, Myoglobin, Tissue plasminogen activator, Fatty acid binding protein-4). The differences can most probably be explained by the differences in statistical analysis, observation time, or the differences in the meal composition between the studies.

There are some limitations that should be addressed. The present investigation studied the effect of food intake only in young healthy Caucasian subjects. Studies are warranted in older healthy subjects, different ethnic groups, and in patients with various cardiac diseases to determine whether the findings in the present investigation are reproducible in such populations. Future investigations should also address the effect of meal size and different diets such as high or low fat [29].

In conclusion, the present investigation shows that several biomarkers that have been used to investigate CVD are affected by food intake, but the effect is predominantly modest. Timing of blood sampling in relation to food intake, therefore, appears not to be a major concern. There are, however, some exceptions. Caution concerning food intake should be taken when investigating Insulin-like growth factor-binding protein 1, Azurocidin, Cystatin-B, Myeloperoxidase, Monocyte chemotactic protein 1, or Myeloblastin.
